# A Roadmap for Automatic Surgical Site Infection Detection and Evaluation Using User-Generated Incision Images

**DOI:** 10.1089/sur.2019.154

**Published:** 2019-09-10

**Authors:** Ziyu Jiang, Randy Ardywibowo, Aven Samereh, Heather L. Evans, William B. Lober, Xiangyu Chang, Xiaoning Qian, Zhangyang Wang, Shuai Huang

**Affiliations:** ^1^ Department of Computer Science and Engineering, Texas A&M University, College Station, Texas.; ^2^ Department of Electrical and Computer Engineering, Texas A&M University, College Station, Texas.; ^3^ Department of Industrial and Systems Engineering, University of Washington, Seattle, Washington.; ^4^ Department of Surgery, University of South Carolina, Columbia, South Carolina.; ^5^ Department of Biobehavioral Nursing and Health Informatics, University of Washington, Seattle, Washington.; ^6^ Center of Data Science and Information Quality, School of Management, Xi'an Jiaotong University, Shaanxi Sheng, China.

**Keywords:** surgical site infection, wound healing, wound management

## Abstract

***Background:*** Emerging technologies such as smartphones and wearable sensors have enabled the paradigm shift to new patient-centered healthcare, together with recent mobile health (mHealth) app development. One such promising healthcare app is incision monitoring based on patient-taken incision images. In this review, challenges and potential solution strategies are investigated for surgical site infection (SSI) detection and evaluation using surgical site images taken at home.

***Methods:*** Potential image quality issues, feature extraction, and surgical site image analysis challenges are discussed. Recent image analysis and machine learning solutions are reviewed to extract meaningful representations as image markers for incision monitoring. Discussions on opportunities and challenges of applying these methods to derive accurate SSI prediction are provided.

***Conclusions:*** Interactive image acquisition as well as customized image analysis and machine learning methods for SSI monitoring will play critical roles in developing sustainable mHealth apps to achieve the expected outcomes of patient-taken incision images for effective out-of-clinic patient-centered healthcare with substantially reduced cost.

The U.S. healthcare sector is transitioning from reactive care to proactive care, with the emphasis shifting to preventive measures and early interventions. Patients are increasingly empowered in this endeavor, both as a goal and as a result. Empowered patients may actively seek ways to engage in their healthcare using emerging technologies such as smartphones and wearable sensors. Recent studies have shown that patients are already using camera phones to e-mail and text incision photos to their providers [1], with access often prompted by providers. As a response to growing technology use among patients, a number of smartphone apps have been developed, such as the mPOWEr app (mobile Post-Operative Wound Evaluator; https://mpowercare.org), that enable patients to monitor their surgical sites for signs and symptoms of surgical site infection (SSI) at home and transmit photographs and self-reported incision and clinical observations to physicians. This generates promising new types of data that may address many challenging SSI problems, whereas the enormous scale of the data and the novelty also bring exciting intellectual challenges requiring close collaboration between medical professionals, statisticians, and computer scientists.

Increasingly available image data, together with other new types of data, naturally inspire the adoption of data-driven methods, such as machine learning, to extract information and make use of information-rich but statistically complex data. However, classic machine learning algorithms often have difficulty extracting semantic features directly from raw data. This phenomenon, commonly known as the semantic gap [2], requires assistance from domain knowledge for hand-crafted feature representations, on which machine learning models operate more effectively. In contrast, more recent deep learning approaches derive semantically meaningful representations, through construction of a hierarchy of features to represent a sophisticated concept. Deep learning requires less hand-engineered features and expert knowledge, and has recently achieved tremendous success in visual object recognition [3–6], face recognition and verification [7,8], object detection [9–12], image restoration and enhancement [13–18], clustering [19], emotion recognition [20], aesthetics and style recognition [21–24], scene understanding [25,26], speech recognition [27], machine translation [28], image synthesis [29], and even playing Go [30] and poker [31].

This promising progress has also motivated the widespread usage of deep learning in the medical image fields. For example, UNet [32] was first applied successfully in medical image segmentation. Esteva et al. [33] trained an end-to-end deep network and achieved dermatologist-level performance in classifying skin cancer. Moreover, deep learning is also applied successfully to many other tasks in the medical image context such as knee cartilage segmentation [34], diabetic retinopathy detection [35–37], lymph node detection [38–41], pulmonary nodule detection [42–44], brain lesion segmentation [45–48], and Alzheimer's disease classification [49–53]. A comprehensive survey can be found in Litjens et al. [54].

Our goal is to borrow strengths and translate the successes achieved those domains in incision image analysis for SSI, and further reinforce those domains with new methods developed to tackling new challenges in SSI. In this article, we propose a roadmap for developing incision image algorithms for automatic SSI detection and evaluation. Challenges persist, ranging from limited photo quality and uncontrolled imaging variations (e.g., light and angle), to the enormous heterogeneity of patients that calls for personalization in our algorithms [55]. We introduce both novelty and challenges in using incision images for SSI detection and evaluation and provide an overview of recent and related developments in computer vision, medical imaging processing, and analysis. We discuss a roadmap that could lead us to a systematic development of computational algorithms to detect and track SSI risk accurately using incision images captured by smartphones in a variety of conditions by a heterogeneous population.

## Challenges in User-Generated Incision Images

As the National Patient Safety Agency has recently reported, 11% of serious medical events leading to mortality or substantial morbidity incidents are a function of unrecognized progression of disease [56,57]. This is particularly true for SSI because its natural history is still largely unknown. Whereas incision image data (and other types of user-generated data) offer great promise in helping clinicians capture disease progression with unprecedented and fine-grained resolution, it is challenging to extract risk-predictive patterns that correlate with the underlying disease progression because of challenges we discuss in the following subsections.

### Quality issues in image acquisition

**FIG. 1.**
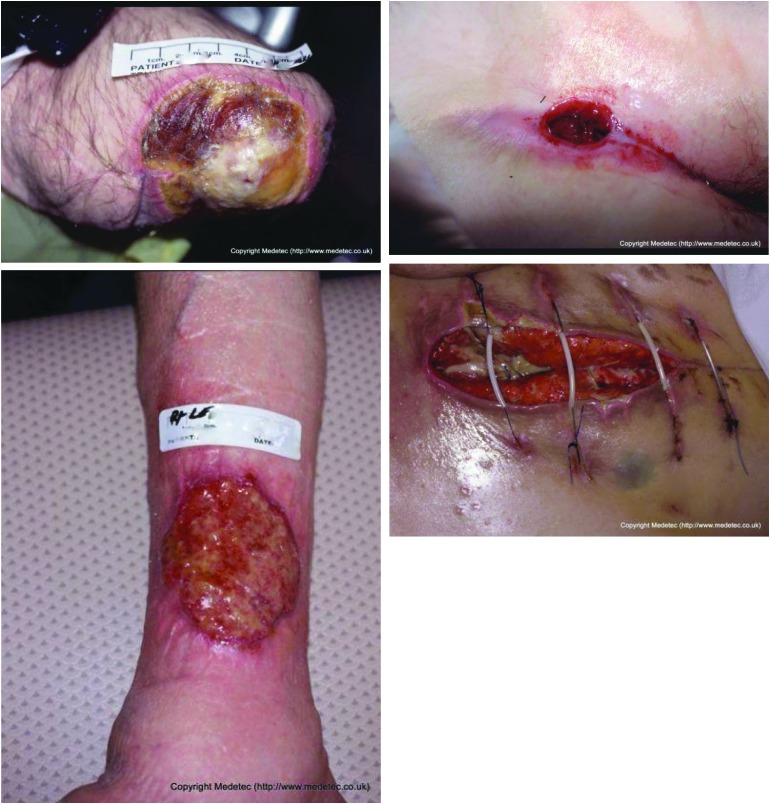
Example of the challenges faced characterizing surgical site images, including poor lighting conditions, obstructing objects such as hair, stitches, and thin films, as well as different camera angles and positioning. Color image is available online.

To extract clinically meaningful features from the user-generated incision images, we have to overcome the challenges presented by images taken by patients or family members who do not have clinical backgrounds and are taken using different types of devices in a variety of naturalistic environments. These challenges have not been addressed adequately in the existing literature. For example, images may be taken under different lighting conditions, and the positioning and size of the incision in the image may change between different images of the same incision (Fig. 1). Frequently, obstructing objects are included in incision images.

Several methods have been proposed to alleviate the issues above, including using apertures to ensure consistent lighting conditions, using transparent films over the incision, and using color or size fiduciaries such as a common object or uniform color template. One goal might be to develop automated guidance for patient–photographers, such as the image guidance provided in check-deposit banking applications, which may prompt the user to move closer or further away to optimize size and focus, or that may give warning of inadequate contrast or lighting. However, even with automated guidance, different healthcare during guidance will produce images with different styles [58]. Depending on whether these image differences are accounted for, these solutions may weaken the monitoring framework. In Shenoy et al. [59], the light condition is consistent in the incision images and incision is always centered. Under these conditions, their network achieved a high F1 score and this indicates the promising performance of available approaches after the quality issue is solved.

### Feature extraction

It is apparent that image data provides an unprecedentedly rich source of data for SSI research, although this information may be masked by the wide variations in image acquisition. Important features extracted from images of the surgical site might include incision size, granularity, color, and morphology [60]. Image processing and analysis algorithms have been developed to extract these features directly from incision images. In addition to these traditional image analysis algorithms focusing on predefined image features, recent deep learning algorithms can automatically find feature representations that would be useful to monitor SSI, allowing for the discovery of new image features indicative of SSI progression [61].

Traditional teaching is that redness (erythema) adjacent to the incision indicates SSI, however, work by Sanger et al. [62] in characterizing the predictive value of provider incision observations of hospitalized patients did not show erythema to be predictive of infection, suggesting the importance of further investigation. There is no universal definition of imaged erythema, which would need to incorporate different skin tones, possibly individual patient responses to infection/inflammation, and imaging condition variability. Systematic characterization would be needed to understand the relation between SSI and either erythema as an extracted image feature, or as a deep learning feature representation of incision images.

### Incision segmentation

To overcome the enormous variations in image acquisition and lay a reliable foundation for effective feature extraction, incision segmentation (spatial identification of the incision within the image) is a critical tool. An immediate goal of incision segmentation is to extract the size information of the incision area for SSI evaluation [63]. Segmentation can also be used to remove complex background distractions. Algorithms for incision segmentation have mostly been developed using incision images captured by skilled professionals with relatively well-controlled experiments, i.e., with consistent use of image acquisition device and procedure on a selected cohort. Early work involved applying a region growing method and automatic selection of the best channel [64] and developing active contour model in which the minimax principle was used adaptively to regularize the contour according to the local conditions in the incision image [65]. However, this method often contained many parameters that required manual adjustment for different images. Moreover, as we target user-generated incision images that come from amateur imaging devices (e.g., hand-held mobile phones), we expect image quality to be both decreased and variable [55]. In addition to the diverse incision characteristics, the imprecise definition of incision boundaries also complicates the problem. There are typically transition regions between incision and normal skin, but there is no clear consensus on image criteria to identify an incision boundary.

## Recent Developments in Computer Vision and Medical Imaging Analysis

### Deep learning

A basic neural network is composed of a set of perceptrons (artificial neurons), each of which maps inputs to output values with a simple activation function. Taking image classification as an example [3], a deep learning-based image classification system represents an object by gradually extracting edges, textures, and structures, from lower to middle-level hidden layers, which becomes more and more associated with the target semantic concept as the model grows deeper. Driven by the emergence of big data and hardware acceleration, the intricacy of data can be extracted with higher and more abstract level representation from raw inputs, gaining more power for deep learning to solve even traditionally intractable problems.

Among recent deep neural network architectures, convolutional neural networks (CNNs) and recurrent neural networks (RNNs) are the two main streams, differing in their connectivity patterns. Convolutional neural networks deploy convolution operations on hidden layers for weight sharing and parameter reduction. Convolutional neural networks can extract local information from grid-like input data and have mainly shown successes in computer vision and image processing, with many popular instances such as LeNet [66], AlexNet [3], VGG [67], GoogLeNet [68], and ResNet [69]. Recurrent neural networks are dedicated to processing sequential input data with variable length. Recurrent neural networks produce an output at each time step. The hidden neuron at each time step is calculated based on input data and hidden neurons at previous time step. To avoid vanishing/exploding gradients of RNNs in long-term dependency, long short-term memory (LSTM) [70] and gated recurrent unit (GRU) [71] with controllable gates are used widely in practical applications.

Recent deep networks have been shown to accomplish many tasks with substantial performance improvements over traditional image processing methods. For example, UNet [32], SegNet [72], ReSeg [73], MaskRCNN [74], and PSPNet [75] have achieved considerable performance progress in image segmentation. ResNet [69], GoogleNet [67], InceptionV3 [76], VGG [77], and NASNet [78] have achieved exceptional performance in image classification. Deep models can also be enhanced with particular robustness to real-world image degradations such as low resolution and noise [4,5,79], and therefore becoming more applicable to non-ideal quality photos from mobile devices. Readers with further interest are referred to the comprehensive deep learning textbook [80].

### Feature extraction from incision images

Feature extraction methods for incision images have relied on identifying interpretable features indicative of incision progression. For example, a prevalent system widely used in incision assessment is the red-yellow-black system to identify granulation, slough, and necrotic tissue types [81]. Most traditional feature extraction methods have relied on first segmenting the incision into these distinct classes and then proceeding to extract features from these classes separately. For example, Mukherjee et al. [82] extracted features such as mean, standard deviation, skewness, kurtosis, and local contrast from 15 different color spaces. Using these features, a support vector machine (SVM) was used to segment the incision images into the red-yellow-black system for tissue type identification.

Many studies have indicated that color features are more useful for incision tissue classification compared with textural features [83–85], showing the effectiveness of the red-yellow-black system. However, textural features could further refine the incision assessment. Kolesnik and Fexa [86] studied the robustness of SVM models that only used color features, in comparison with SVM models that used both color and textural features for incision segmentation. Their study indicated that the textural features reduced the average magnitude of segmentation error compared to using only color features.

Moreover, color correction of the incision images has been shown to make feature extraction more robust. A study done by Wannous et al. [85] decomposed color correction into two distinct problems by obtaining a consistent color response via adjusting camera settings first and then determining the relation between the device-dependent color data and the device-independent color data. They conquered the second problem by placing a small Macbeth pattern in the camera field and achieved an improvement in accuracy from 68% to 76%.

### Incision segmentation

As mentioned earlier, incision segmentation would be a critical tool to facilitate and enhance feature extraction from incision images. Particularly, machine learning-based methods [87] for incision segmentation have been found particularly promising to achieve full-automation and self-adaption. Early works include the use of SVM classifier, e.g., as in Kolesnik and Fexa [84], by treating incision segmentation as a binary classification task. Kolesnik and Fexa [86] further evaluated the robustness of SVM for incision segmentation. Their research indicates that it is not stable for new incision images and therefore not feasible for an automatic system. In addition, neural networks, Bayesian classifiers, and random forest decision trees were also utilized [88,89]. However, these methods relied highly on the choice of hand-crafted features that were created based on prior knowledge, which could only explore a limited amount of the image information.

**FIG 2.**
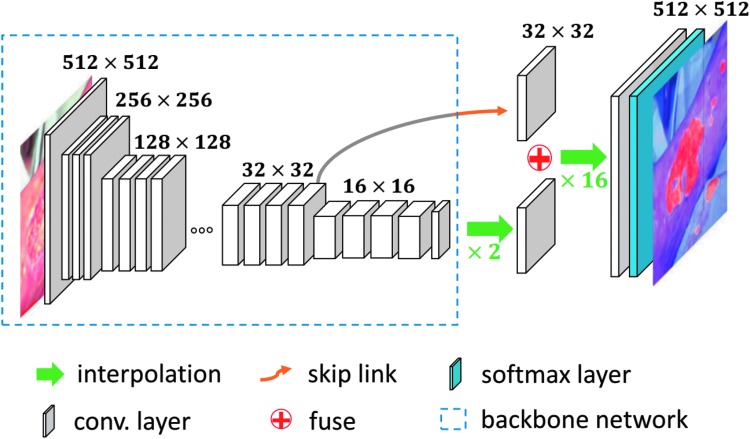
The architecture of WoundSeg [63]. Color image is available online.

The recent advance of deep learning brought tremendous developments to incision image processing, although many of them were not developed for user-generated incision image data. Since Long et al. [90] proposed fully convolutional networks (FCN), which extended the successful deep learning classification framework to segmentation task by replacing the fully connected layers with convolutional layers, many incision segmentation models were developed based on it. These models could be trained end-to-end and achieve superior performance. An encoder–decoder was utilized for the segmentation of incision by Lu et al. [91] and Wang et al. [92]. The WoundSeg, with higher performance and efficiency, was proposed by Liu et al. [63]. The architecture of the WoundSeg is shown in Figure 2. It modifies the FCN [90] structure by adding a skip connection from previous feature map with higher resolution for making the segmentation result finer. They also leverage data augmentation and post-processing, which together improve the accuracy to 98.12%. However, their dataset is taken by a professional in a hospital environment and the quality of images is ensured.

Because all these methods used supervised learning that required a lot of expert labeled data, it is an expensive approach if the labeling cost is considerable. Many studies also explored unsupervised methods such as clustering in incision segmentation. Yadav et al. [93] compared k-means and fuzzy c-means on Dr and Db color channels. The spectral approach based on the affinity matrix was explored in Dhane et al. [94]. Dhane et al. [55] further proposed fuzzy spectral clustering (FSC) that constructed similarity matrix with gray-based fuzzy similarity measure using spatial knowledge of an image. How scalable and practical these methods for analyzing user-generated incision images to tackle SSI problems demands many further studies and validations.

## Discussion

With the rapid development of machine learning and medical image analysis, many developing techniques can potentially be adopted to address the existing challenges. In this section, we discuss some of the possibilities.

### Learning-based image processing and enhancement

As we have elaborated earlier, incision images taken by amateur users are, in general, of low quality compared with professional skin scans. Importantly, they would display huge variations of lighting conditions, e.g., because of shadows or over-/underexposures, which will greatly jeopardize both feature extraction and incision region segmentation. Many deep learning based enhancement algorithms, which are trained to regress low-quality images to enhanced versions, could potentially be used. In other words, it is to map those low-quality images into high-quality incision image templates created by incision image data collected by professionals such that the low-quality image could be automatically calibrated, de-noised, enhanced, and imputed. Although this is a promising idea, more challenges arise from the fact that in the field of incision image analysis, there currently does not exist, nor will it be easy to collect, a large set of paired low-quality/enhanced images for training such models.

**FIG 3.**
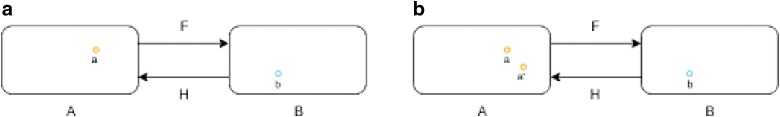
(**a**) Summer picture and (**b**) corresponding winter picture while F and H are translation functions. (**b**) Consistency loss measures the distance between a and a 0, in which a 0 = H(F(a)). Color image is available online.

**FIG 4.**
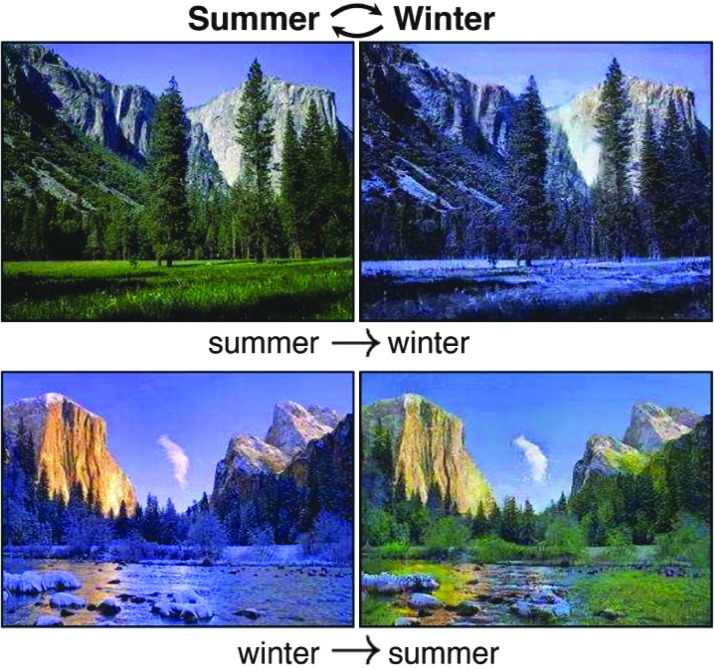
The domain translation between summer and winter pictures [95]. Color image is available online.

A tentative solution is to treat this as an image domain translation problem, in which domain is formed by a set of images with the same pattern. For example, landscape pictures taken in summer and winter can be treated as from two domains. The summer pictures are from domain A and the winter pictures are from domain B as shown in Figure 3(a). For each summer picture a from domain A, there exists a corresponding winter picture b in domain B, and thus, a can be transformed to b through a function F. The function F is called as domain translation function and the function H is the domain translation function from B to A. A translation demonstration between them is shown in Figure 4. Whereas in many applications, the reality is that there is no paired sample from domain A and B, we could resort to optimization solutions in a statistical framework. For example, as shown in Zhu et al. [95], a novel consistency loss was developed to measure the distance of the sample a and a 0 = H(F(a)) as shown in Figure 3(b). For good domain translation functions F and H, the distance between a and a 0 should be small. Therefore, with unpaired data from two domains, we can optimize the translation functions through minimizing the consistency loss. Similar techniques were lately applied to natural image enhancement applications [96], and we expect the idea to also be helpful for incision image enhancement.

### Interactive image capture

An important question is whether smartphone-based systems have any disadvantage in image capture quality compared with traditional medical workstations. Kumar et al. [97] showed that teleophthalmology images taken by a smartphone had a near-identical quality to images taken by a standard medical workstation. This suggests that images captured using smartphones do not suffer from any major quality issues compared with those taken using medical workstations, as long as sufficient quality control is put in place.

A solution that can potentially increase the quality of SSI images acquisition is to interactively guide the user when taking the SSI images. Indeed, a system that aids patients and medical personnel in adjusting/finding correct lighting conditions, exposure, and incision location could solve most of the image quality issues we highlighted previously. Such a system not only needs to have sufficient quality control mechanisms to obtain accurate SSI incision features, but also needs to be user-friendly. Recent medical image capture systems have utilized mobile phone-based applications to facilitate image capture, such as in teleophthalmology, clinical microscopy, and diabetic wound treatment [97–99]. Such interactive image capture methods have many notable advantages. Agu et al. [99] noted that the use of smartphones for medical image capture had the benefits of easy deployment as smartphone applications are easily developed and installed, and accessibility as smartphones are conveniently available to any person. In addition, new hardware can be timely leveraged to help SSI monitoring because smartphone hardware is upgraded frequently.

For the case of SSI image capture, existing incision assessment systems have relied on using peripheral or ancillary devices to control the lighting and incision positions. Moreover, thin films that overlay graph paper with mesh grids are typically used when measuring incision size [100–102]. An interactive incision image capturing system would need to have these mechanisms in place to ensure proper image quality.

**FIG 5.**
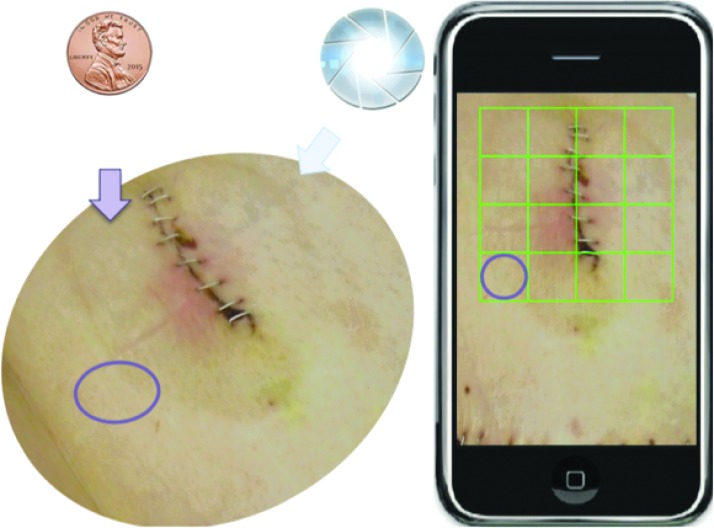
Interactive procedure for image capture using mobile phones. Color image is available online.

**FIG 6.**
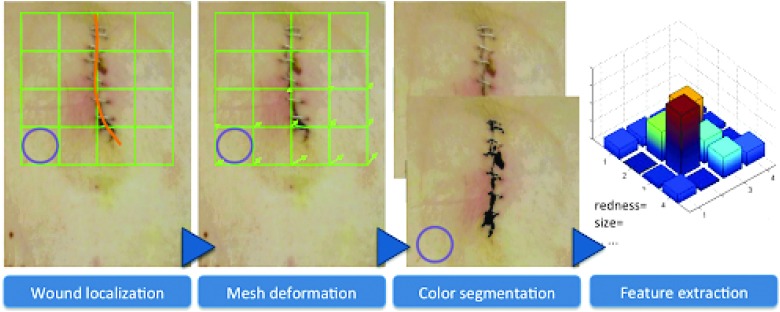
Envisioned interactive image capture system. Color image is available online.

Such an interactive system can be shown in Figures 5 and 6. As shown in Figure 5, a coin is placed near the incision image as a size indicator, while a virtual mesh grid is used to replace the graphpaper thin film traditionally used. After the picture is taken, the incision is localized as shown in Figure 6. The user is asked to draw the contour of the incision region to allow a deformation of the mesh grid to normalize the incision region size. With this normalized image, color segmentation can be conducted, and interpretable feature of the incision can be extracted. Lighting conditions can also be normalized, and features can be readily extracted from the SSI images for effective and reliable SSI monitoring.

### Incision image segmentation and assessment

As mentioned earlier, challenges in segmenting and assessing incision images using deep learning center around three main issues: the labeled data volume, the segmentation label quality, and the interpretability and domain knowledge integration.

To overcome the first challenge, we resort to data augmentation, a widely utilized tool in deep learning to artificially increase the labeled data volume and diversity, without extra data collection efforts [3]. The common means relies on identifying label preserving transformations, i.e., variations known to exist in real data but not affecting semantic annotations. Moreover, models trained with such augmented data will also gain invariance to the selected types of variations. For example, one can alter the lighting conditions as well as color tones of labeled incision images for robust feature learning to varying lighting and skin color. For example, following Wang et al. [21], we can apply γ correction to the luminance channel with random γ values; a gradient in illumination could be further added to simulate an oriented light source. The lighting augmentation will not affect either image or pixel-level annotations.

**FIG 7.**
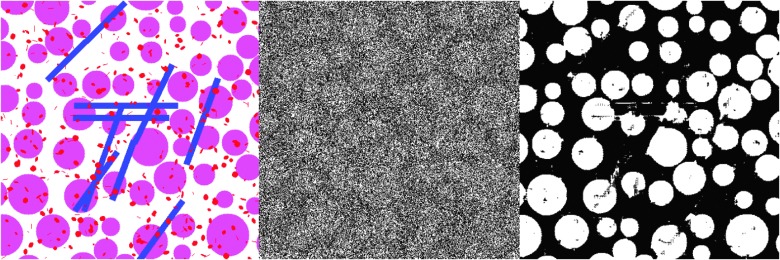
Deep image segmentation with noisy labels: (**left**) synthetic testing image example; (**middle**) simulated noisy segmented image; (**right**) image segmentation by modified U-net using the simulated noisy segmentation. Color image is available online.

The second challenge of segmentation label quality results from the fact that pixel-wise annotations are often generated using semi-automatic methods such as the watershed algorithm; hence there could be incorrectly labeled pixels, making the supervision information for the segmentation task “noisy.” Motivated by recent success of training deep models with noisy labels [103,104], we could adopt the bootstrap strategy of Reed et al. [103] and introduce a noise layer into the deep image segmentation model as done by Sukhbaatar et al. [104] when we use noisy segmentation supervision. Our preliminary result [107] has shown promising potential of this approach, i.e., we have modified the U-net with a noise layer, which can take noisy segmentation results (Fig. 7, middle) for training and segment synthetic images (Fig. 7, left). Figure 7 (right) shows that such a modification can achieve promising segmentation results.

Eventually, another crucial capability for deep learning models to address SSI is how to make their predictive results interpretable to human. In this regard, many recent works from the interpretable deep learning fields show promise. For example, sensitivity analysis [108] tried to explain a prediction based on the model's locally evaluated gradient (partial derivative). Matching similar image parts could also be a promising method as done by Chen et al. [61]. We refer the readers to a literature review by Chakraborty et al. [107]. Different from existing incision image assessment methods that were either rule-based [100,108,109] or data-driven [63], we advocate to draw the complementary power of knowledge-based feature design and data-driven feature learning to maximize the information extraction from the incision images with robust performances.
